# Opposing functions of white matter in glioblastoma

**DOI:** 10.1093/neuonc/noaf266

**Published:** 2025-11-13

**Authors:** Daniel J Silver, Gunnar H D Poplawski, Justin D Lathia

**Affiliations:** Department of Cancer Sciences, Cleveland Clinic Research, Cleveland, Ohio, USA; Department of Pathology, Cleveland Clinic Lerner College of Medicine, Cleveland, Ohio, USA; Case Comprehensive Cancer Center, Case Western Reserve University, Cleveland, Ohio, USA; Rose Ella Burkhardt Brain Tumor & Neuro-Oncology Center, Cleveland Clinic, Cleveland, Ohio, USA; Department of Cancer Sciences, Cleveland Clinic Research, Cleveland, Ohio, USA; Department of Molecular Medicine, Cleveland Clinic Lerner College of Medicine, Cleveland, Ohio, USA; Case Comprehensive Cancer Center, Case Western Reserve University, Cleveland, Ohio, USA; Rose Ella Burkhardt Brain Tumor & Neuro-Oncology Center, Cleveland Clinic, Cleveland, Ohio, USA; Department of Cancer Sciences, Cleveland Clinic Research, Cleveland, Ohio, USA; Department of Molecular Medicine, Cleveland Clinic Lerner College of Medicine, Cleveland, Ohio, USA; Case Comprehensive Cancer Center, Case Western Reserve University, Cleveland, Ohio, USA; Rose Ella Burkhardt Brain Tumor & Neuro-Oncology Center, Cleveland Clinic, Cleveland, Ohio, USA

For almost 90 years, we have recognized that myelinated fiber tracts represent preferred migration and growth zones for glioblastoma (GBM) tumors,^1^ yet the biology of the white matter niche remains far less understood than the peri-vascular or hypoxic niches. A recent study, published in *Nature* by Parrinello and Hill’s groups at University College London, titled *Axonal Injury is a Targetable Driver of Glioblastoma Progression*, addresses the white matter niche with rigorous and innovative investigation. Led by Melanie Clements et al,^2^ the work reveals how degenerating white matter accelerates GBM development, particularly during early disease stages.

The team began by examining 4 patient-derived GBM models and a genetically engineered mouse model.^3^ Across these diverse systems, tumors displayed a consistent biphasic growth pattern. In the initial phase, GBM cells infiltrated forebrain white matter. Cancer pervaded the corpus callosum, anterior commissure, and striatal fiber tracts but preserved overall brain architecture. Ki67 staining indicated minimal proliferation, and astrocytes and myeloid cells were only modestly activated. Gliotic barriers were thin, perhaps a single astrocytic process thick, unlike the dense laminations typical of injury-associated glial scars. This early stage lacked mass effect or anatomical distortion, suggesting that the white matter niche imposes certain constraints on tumor expansion.

Later, tumors escaped these constraints, entered a phase of rapid proliferation, and developed into the bulk disease that in the human brain drives symptom presentation and clinical detection. This transition coincided with architectural breakdown and massive expansion of the tumor bulk. These observations suggested that white matter integrity restrains GBM growth and that its degeneration may serve as a tipping point for explosive progression.

To test this hypothesis, the authors performed anatomically precise axotomies of the subcortical white matter. Mechanical disruption abolished the slow initial phase, enabling tumors to bypass early containment and immediately enter bulk expansion. Survival times shortened, and transient astrogliosis appeared which was quickly suppressed. These results strongly support the conclusion that white matter degeneration accelerates GBM progression. Whether axon loss alone or accompanying glial and immune changes drive this effect remains unresolved, but the authors argue axon degeneration was the key stimulus.

To preserve axon integrity and test GBM progression in a model where axonal dieback was held to a minimum, the team initiated genetically engineered, endogenously arising GBM in the brains of SARM1 knockout mice (SARM1^−/−^). SARM1 (or Sterile Alpha and TIR Motif Containing 1) is a critical driver of axon dieback, also called Wallerian Degeneration. The investigators noted that SARM1 has become an attractive target for pharmacologic inhibition intended to preserve neuron architecture and function in the hopes of staving off a variety of neuro-degenerative conditions. In SARM1^−/−^ brains, axon integrity was maintained, astrogliosis remained low, and innate immune activation was delayed until neurological endpoints. Strikingly, survival improved by ∼25% and tumors never transitioned to the mass-building phase. Instead, they resembled gliomatosis cerebri, diffusely stippling multiple forebrain regions without causing mass effect or anatomical distortion. Even at endpoint, the neural architecture remained intact. Thus, axon preservation did not halt GBM but locked it within the white matter niche, limiting proliferation and preventing architectural collapse.

These findings suggest neuron degeneration promotes GBM progression, though the biology may be more complex. SARM1 deletion dampened multiple processes: axon loss, glial reactivity, as well as immune activation, making it difficult to isolate the dominant driver. Nonetheless, the work elegantly demonstrated that axonal integrity influences GBM behavior and highlights SARM1 inhibition as a potential therapeutic strategy.

Several critical questions remain. The study assumes early-stage GBM is more treatable, yet evidence for this is lacking. Most GBM tumors are detected late, after bulk expansion and widespread invasion. Whether axon-preserving interventions help at these stages is unknown. Additionally, all experiments used young mice, whereas GBM typically arises in aged brains^4–6^ where baseline neurodegeneration may precede tumorigenesis. Age-related neuron loss may diminish the role of axon dieback and challenge the utility of SARM1 inhibition. Future studies must address whether age-related changes alter the contribution of axon dieback to GBM progression.

Prior research supports an inhibitory nature of white matter. Amberger et al^7^ showed GBM must proteolyze white matter to invade; Hong et al^8^ linked invasion to the downregulation of the NOGO receptor; and Parrinello’s^9^ own work demonstrated that the white matter niche exerts a differentiating pressure on GBM cells. Clements et al now add compelling evidence that axon damage accompanies early GBM growth and may be targetable. Whether degeneration itself or the tumor’s mechanisms to overcome white matter inhibition drive progression remains unresolved.

In total, this study combines meticulous histopathology, spatial transcriptomics, transplantable, and genetic models to propose axonal injury as a modifiable factor in GBM biology. While therapeutic implications require validation, especially in aged brains and late-stage disease, the work refocuses our attention on the white matter niche as a dynamic player in gliomagenesis and opens new avenues for intervention. Like the best research, it raises more questions than it answers, challenging us to rethink how microenvironmental integrity shapes tumor progression.

## Conflict of Interest Statement

D.J.S. and G.H.D.P. have no conflicts of interests related to the topic of this manuscript. J.D.L. is listed as an inventor on several cancer therapeutic patents held by the Cleveland Clinic, but these are not relevant to the topic of this manuscript.

## Funding

No direct funding was used to support the generation of this manuscript.

## Data Availability

No new data were generated or analyzed to generate this editorial.

## References

[1] Scherer HJ. The forms of growth in gliomas and their practical significance. Brain. 1940; 63:1–35.

[2] Clements M , Tang W, Baronik ZF, et al Axonal injury is a targetable driver of glioblastoma progression. Nature. 2025;646:452–461. 40836081 10.1038/s41586-025-09411-2 PMC12507684

[3] Clements M , Ragdale HS, Garcia-Diaz C, Parrinello S. Generation of immunocompetent somatic glioblastoma mouse models through in situ transformation of subventricular zone neural stem cells. STAR Protoc. 2024;5:102928. 38430519 10.1016/j.xpro.2024.102928 PMC10914519

[4] Ostrom QT , Gittleman H, Fulop J, Liu M, Blanda R, Kromer C, Wolinsky Y, Kruchko C, Barnholtz-Sloan JS. CBTRUS statistical report: primary brain and Central nervous system tumors diagnosed in the United States in 2008-2012. Neuro Oncol., 2015:iv1-iv62. 26511214 10.1093/neuonc/nov189 PMC4623240

[5] Ladomersky E , Zhai L, Lauing KL, et al Advanced age increases immunosuppression in the brain and decreases immunotherapeutic efficacy in subjects with glioblastoma. Clin Cancer Res. 2020;26:5232–5245. 32546647 10.1158/1078-0432.CCR-19-3874 PMC7541490

[6] Kim M , Ladomersky E, Mozny A, et al Glioblastoma as an age-related neurological disorder in adults. *Neuro-Oncol Adv*. 2021;3:vdab125. 10.1093/noajnl/vdab125 PMC8500689 34647022

[7] Amberger VR , Hensel T, Ogata N, Schwab ME. Spreading and migration of human glioma and rat C6 cells on central nervous system myelin in vitro is correlated with tumor malignancy and involves a metalloproteolytic activity. Cancer Res. 1998;58:149–158. 9426071

[8] Hong JH , Kang S, Sa JK, et al Modulation of nogo receptor 1 expression orchestrates myelin-associated infiltration of glioblastoma. Brain. 2021;144:636–654. 33479772 10.1093/brain/awaa408

[9] Brooks LJ , Clements MP, Burden JJ, et al The white matter is a pro-differentiative niche for glioblastoma. Nat Commun. 2021;12:2184. 33846316 10.1038/s41467-021-22225-w PMC8042097

